# Cecal volvulus following appendectomy in a teenage patient: A case report

**DOI:** 10.1002/ccr3.8480

**Published:** 2024-02-06

**Authors:** N. Khanal, R. Subedi, N. Shrestha, S. B. Pradhan, P. Shah, S. Shrestha, S. Wagle

**Affiliations:** ^1^ Department of Surgery Hetauda Hospital, Madan Bhandari Academy of Health Sciences Hetauda Nepal; ^2^ Department of Radiology Hetauda Hospital, Madan Bhandari Academy of Health Sciences Hetauda Nepal

**Keywords:** abdominal pain, acute appendicitis, appendectomy, Cecal volvulus, Cecopexy, vomiting

## Abstract

Patients mimicking appendicitis symptoms in a rural setting or those post‐appendectomy, indicating cecal volvulus, should always be considered. Swift action can prevent catastrophic consequences.

**Abstract:**

We present a case of a 14‐year‐old female who initially underwent open appendectomy for acute appendicitis and subsequently experienced symptoms of abdominal distention, vomiting, and fever. Her condition deteriorated following the appendectomy, despite a prior appendectomy for similar symptoms at a different facility. A computed tomography (CT) scan identified cecal volvulus as the underlying issue. This led to the performance of a laparotomy, cecopexy, and decompressive ileostomy. After six weeks, ileostomy closure was successfully carried out, and the patient currently enjoys good health. This case highlights the significance of considering uncommon factors as potential contributors to postoperative complications in young patients.

## Graphical abstract

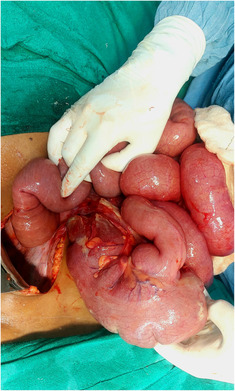

## 1 INTRODUCTION

Cecal volvulus is a highly uncommon yet critical medical condition marked by the abnormal rotation of the cecum and the ascending colon around the mesentery.^1^, ^2^ It is a relatively rare but serious condition that can have severe consequences, including severe abdominal pain, bloating, and vomiting. Left untreated, this can lead to tissue ischemia, gangrene, and even perforation in the affected bowel posing a life‐threatening risk.^3^

In this report, we present a case of cecal volvulus in a 14‐year‐old female patient who initially exhibited symptoms that closely resembled those of acute appendicitis. This case underscores the diagnostic challenge and clinical significance of recognizing cecal volvulus, even when it initially presents as a different condition, to ensure timely intervention and prevent potentially catastrophic outcomes.

## 2 CASE PRESENTATION

A 14‐year‐old female from a rural area presented with complaints of right iliac fossa (RIF) pain, fever, and vomiting two‐day duration. Physical examination revealed localized tenderness in the RIF with guarding. Laboratory investigations showed leukocytosis (white blood cell count: 16,000/mm^3^). The urine pregnancy test was negative; the Ultrasound abdomen was inconclusive revealing probe tenderness in RIF. With a clinical diagnosis favoring acute appendicitis, the patient underwent an emergency open appendectomy, during which an inflamed appendix was removed. However, her postoperative course was marked by persistent abdominal pain, increasing fever, and a progressive deterioration of her overall condition. She was then referred to us. Upon admission and following resuscitation, a contrast‐enhanced computed tomography (CT) scan was performed to evaluate her condition further.

**FIGURE 1:**
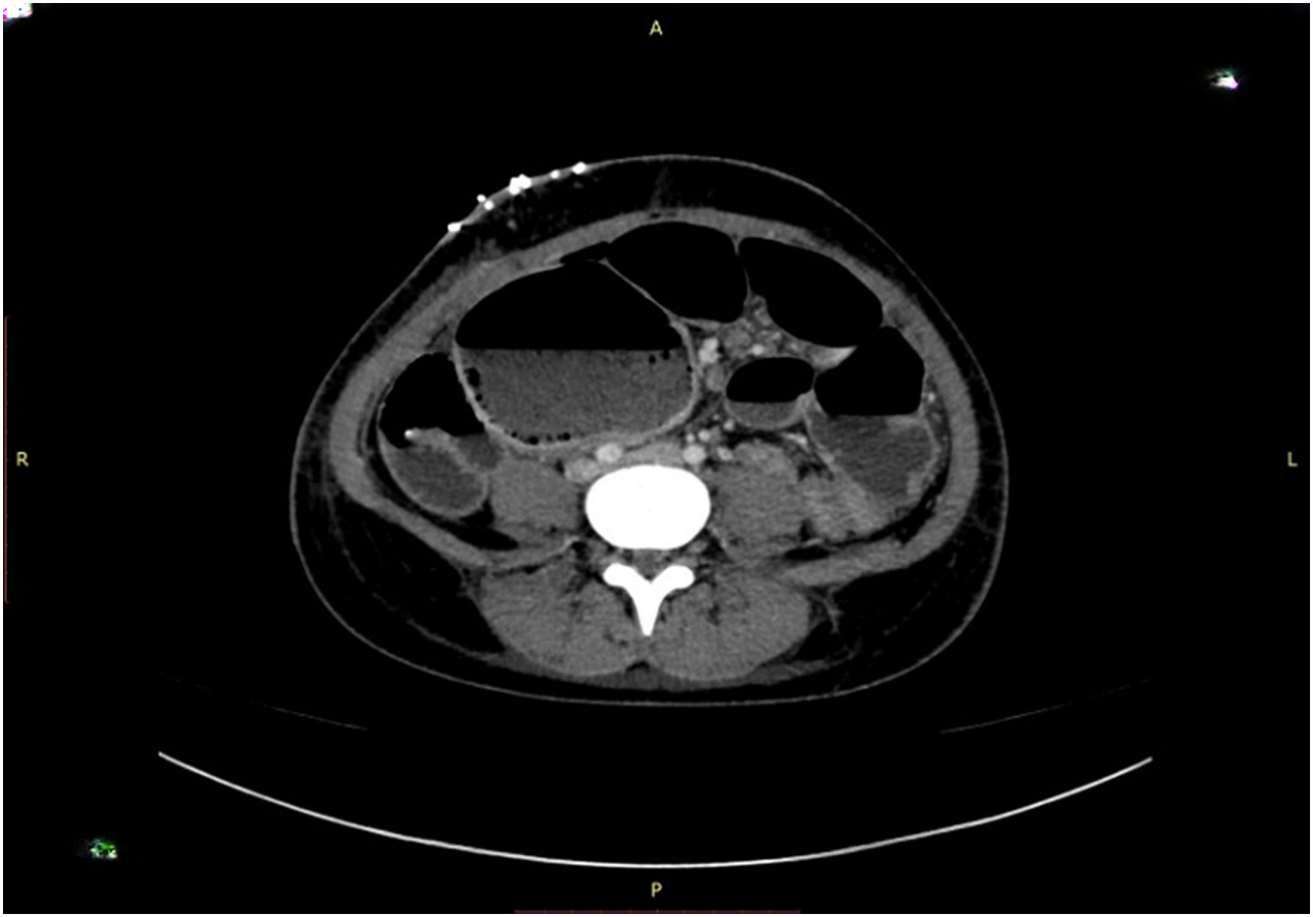
Axial view showing dilated bowel loops with air fluid level. Dilated bowel loops show mottled appearance with small bowel feces sign suggestive of obstruction with appendectomy clip in situ.

**FIGURE 2:**
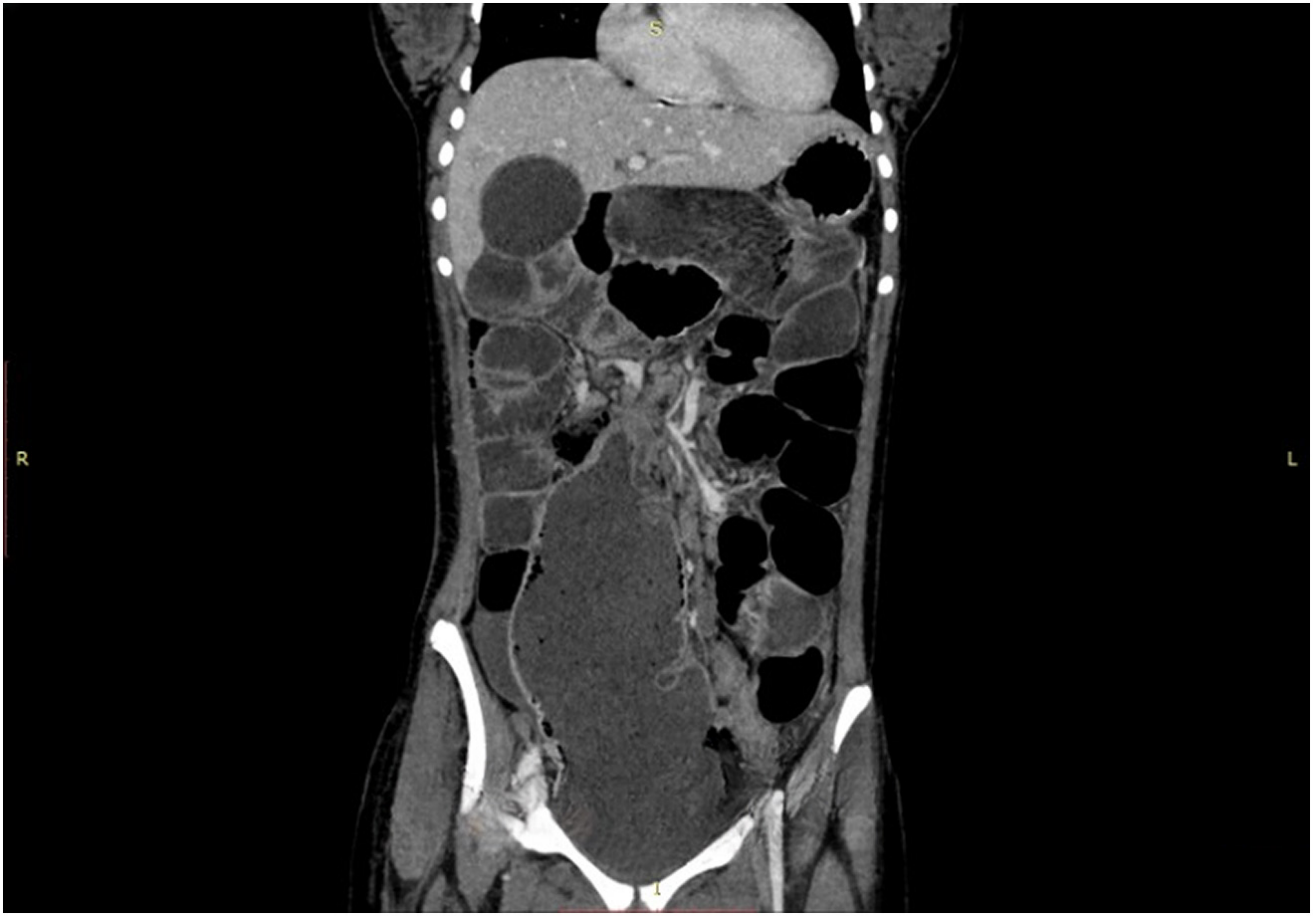
Coronal image showing focal beak like protrusion indicating the site of twisting.

The CT scan **(**Figure 1 and Figure 2 **)** revealed cecal volvulus, with the cecum and ascending colon twisted around their mesentery, causing bowel obstruction. In response to these findings, an urgent laparotomy was performed.

**FIGURE 3:**
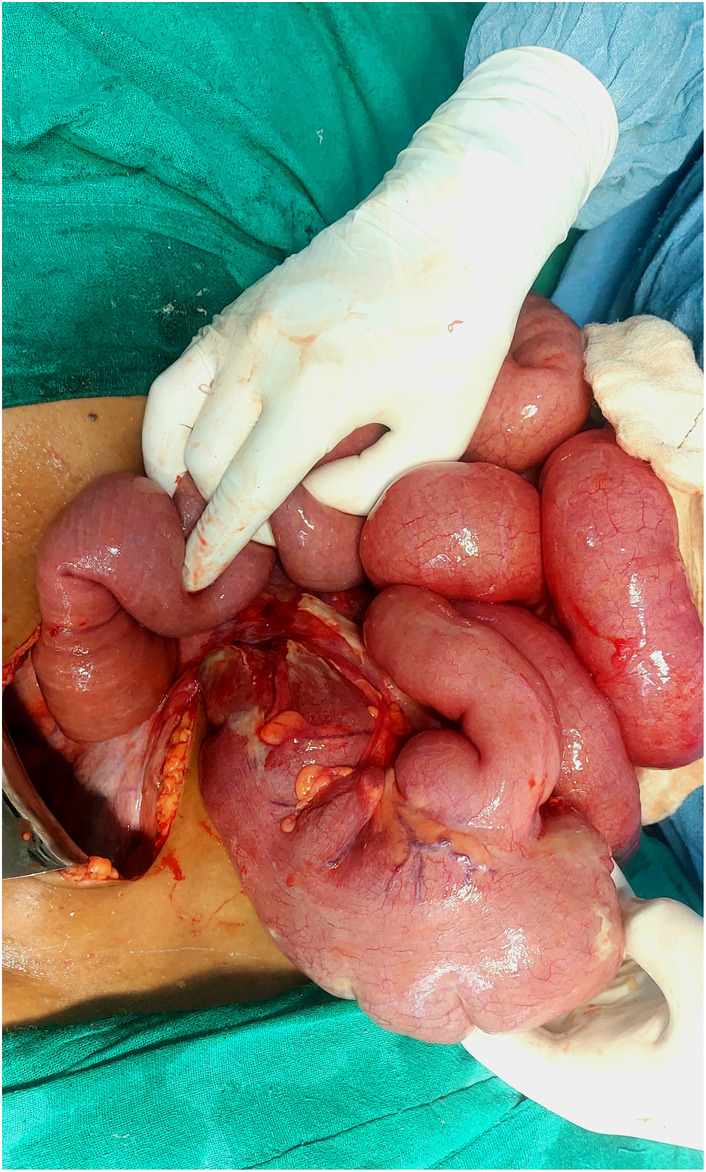
Intra Operative photograph showing twisted and distended caecum.

**FIGURE 4:**
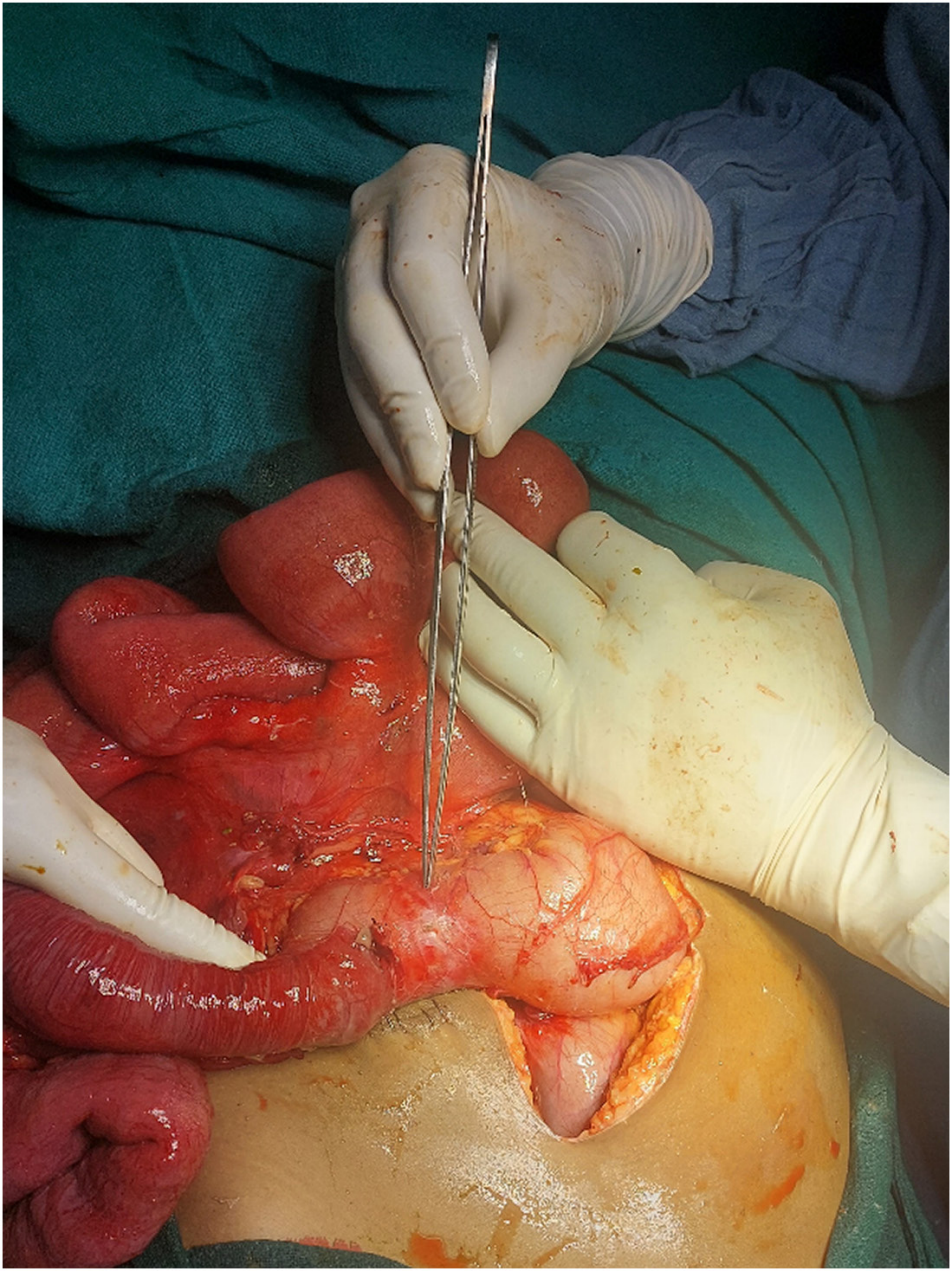
Intra Operative photograph showing distended ileum, ileocolic junction, and caecum following detorsion of caecum.

During the laparotomy, the cecum and ascending colon were found to be twisted counterclockwise around their mesentery (Figure 3) causing a closed‐loop obstruction. Detorsion of the volvulus was attempted but proved difficult, decompressive loop ileostomy was done, bowel decompressed and cecopexy was done (Figure 4). Postoperatively, the patient received broad‐spectrum antibiotics, bowel rest, and close monitoring in the intensive care unit. Her recovery was marked by a gradual improvement in her clinical condition, normalization of laboratory parameters, and return of bowel function.

**FIGURE 5:**
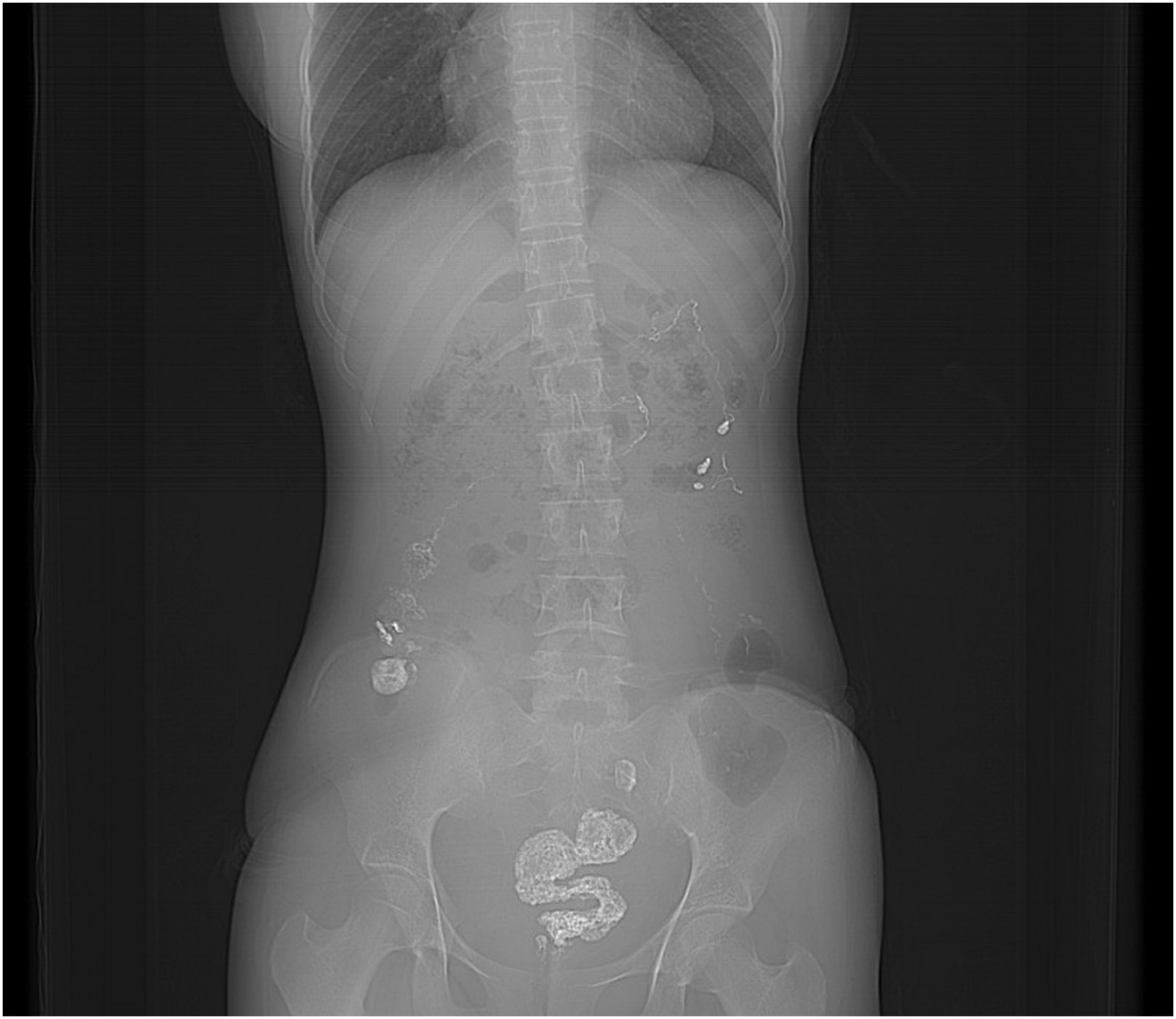
Scout image showing column of contrast in the Right iliac fossa region. The contrast is seen passing till the level of rectum suggestive of patent bowel.

Six weeks later a CT Loopogram (Figure 5) was done, following which the patient underwent ileostomy closure. This procedure was uneventful, and she recovered well in the postoperative period. The patient has since remained asymptomatic and has returned to her normal daily activities.

## 3 DISCUSSION

Cecal volvulus is a condition in which the cecum and ascending colon are twisted axially around the mesentery and vascular pedicles.^4^ Typically, it affects older adults with colonic redundancy, a condition in which the colon features additional loops or twists, making it more susceptible to torsion. However, encountering cecal volvulus following an appendectomy, especially in a pediatric patient, as in our case, is even rarer.

The pathogenesis of cecal volvulus is multifactorial and may involve congenital abnormalities, the presence of redundant colon loops, or post‐surgical adhesions.^5^ The clinical signs and symptoms can vary greatly including common symptoms like severe abdominal pain, constipation, nausea, vomiting, and abdominal distension.^6^ Prompt diagnosis is of utmost importance because delayed recognition and treatment can lead to severe consequences such as bowel ischemia, gangrene, and bowel perforation. Although cecal volvulus is indeed a rare occurrence among adolescents, high clinical suspicion should be maintained, when a patient present with right lower abdominal pain and an unconventional postoperative recovery.

Abdominal X‐rays can provide a diagnosis for cecal volvulus if three common signs are evident: cecal dilation, a sole air‐fluid level in the lower right abdomen, and the absence of gas in the colon. Nonetheless, around 30% of patients may not exhibit these typical radiographic features.^7^ When it comes to diagnosing acute cecal volvulus, CT scans are now preferable over barium enemas.^8^ The presence of the whirl, ileocecal twist, distal colon decompression, and cecal distention larger than 10 cm are all indicators of cecal volvulus on CT scans.^9^ Effective management of cecal volvulus is surgical intervention which includes simple manual detorsion, rectopexy, caecopexy, cecostomy, and resection (limited or right hemicolectomy) which is mandatory for gangrene and a grossly distended, thin‐walled cecum. Resection can be performed by open or laparoscopic approaches.^10^

Additionally, highlighting the importance of considering cecal volvulus as a potential diagnosis in young patients with abdominal pain, especially following an appendectomy, can raise awareness and understanding of this condition and its potential risk factors among healthcare providers. This, in turn, can aid in the early diagnosis and more timely management of similar cases in the future, potentially preventing life‐threatening complications.

## 4 CONCLUSION

This report emphasizes the importance of a thorough and vigilant healthcare approach, particularly in resource‐limited environments where CT scans may not be accessible. In such cases, surgeons rely on clinical diagnosis to provide treatment. Unforeseen and atypical conditions should be taken into account both before and after surgery. Although cecal volvulus is a rare occurrence, prompt recognition and management of it can lead to positive outcomes, as illustrated in our case.

## AUTHOR CONTRIBUTIONS

**N. Khanal:** Conceptualization; formal analysis; investigation; methodology; project administration; resources; supervision; writing – original draft; writing – review and editing. **R. Subedi:** Project administration; software; supervision; writing – original draft; writing – review and editing. **N. Shrestha:** Conceptualization; formal analysis; investigation; project administration; supervision; writing – review and editing. **S. B. Pradhan:** Conceptualization; methodology; project administration; supervision. **P. Shah:** Formal analysis; project administration; software; supervision. **S. Shrestha:** Formal analysis; project administration; software; supervision; writing – original draft; writing – review and editing. **S. Wagle:** Investigation; resources; software.

## FUNDING INFORMATION

None.

## CONFLICT OF INTEREST STATEMENT

All authors declare that they have no conflicts of interest.

## CONSENT

Since the patient is a child under 16 years of age, written consent was obtained from the parent.

## Data Availability

The data that support the findings of this study are available on request from the corresponding author. The data are not publicly available due to privacy or ethical restrictions.
